# Feet/Footwear-Related Fall Risk Screening Tool for Older Adults: Development and Content Validation

**DOI:** 10.3389/fpubh.2021.807019

**Published:** 2022-02-02

**Authors:** Mariana Wingood, Elizabeth Peterson, Christopher Neville, Jennifer L. Vincenzo

**Affiliations:** ^1^Department of Rehabilitation and Movement Science, University of Vermont, Burlington, VT, United States; ^2^Department of Occupational Therapy, University of Illinois at Chicago, Chicago, IL, United States; ^3^Department of Physical Therapy Education, SUNY Upstate Medical University, Syracuse, NY, United States; ^4^Department of Physical Therapy, University of Arkansas for Medical Sciences, Fayetteville, AR, United States

**Keywords:** injury prevention, falls, older adults, balance, STEADI

## Abstract

**Background and Purpose:**

Screening for feet- and footwear-related influences on fall risk is an important component of multifactorial fall risk screenings, yet few evidence-based tools are available for this purpose. We developed the *Screening Tool for Feet/Footwear-Related Influences on Fall Risk* to support interprofessional health care providers in their efforts to screen for feet/footwear-related influences on fall risk among community-dwelling older adults identified at risk for falling.

**Materials and Methods:**

The study consisted of two phases. During Phase 1, results of a systematic review of lower-limb factors associated with balance and falls informed tool development. The tool's initial draft was evaluated by an external group of nine interprofessional content experts. After incorporating changes recommended by Phase 1 participants, Phase 2 was initiated. During Phase 2, eight new interprofessional experts (19.3 average years of experience) completed the three rounds of a modified Delphi study.

**Results:**

Phase 1 experts recommended modifying eight items and rated the tool's clarity, appeal and clinical feasibility as 81.2/100, 79.1/100, and 76.1/100, respectively. Phase 2 participants suggested combining items with similar recommended actions, adding a question about orthoses, and increasing the specificity of nine items. The refinements resulted in a 20-item screening tool. Each item was approved by the Phase 2 participants with > 80% agreement after two rounds of consensus voting, reflecting the tool's high face and content validity.

**Conclusion:**

The new screening tool has high face and content validity and supports identification of feet- and footwear-related influences on fall risk among community-dwelling older adults. The tool can be used by interprofessional healthcare providers completing a multifactorial fall risk screening on community-dwelling adults identified as being at risk for falling.

## Introduction

The Center for Disease Control (CDC) reported 35.6 million falls among older adults in 2018 (1). Of these, 8.4 million required medical care (1). Additional consequences of falling include functional decline, disability, psychological sequelae (including fear of falling and depression), reduced quality of life, mortality, and higher healthcare costs (2, 3). According to Stevens and Lee (4), 9,563–45,164 medically treated falls could be prevented annually, with an associated Medicare cost reduction of US$ 94 million to US$ 442 million. Together, these facts demonstrate the importance of reducing falls among older adults.

Per the American Geriatric Society and British Geriatric Society (AGS/BGS) guidelines for preventing falls in older adults (5), all older individuals should be asked whether they have fallen in the past year. Further, older persons who present for fall-related medical attention, report recurrent falls in the past year, or report difficulties with walking or balance (with or without activity curtailment) should be identified as at-risk and have a multifactorial fall risk assessment (5). To support healthcare providers in their efforts to screen older adults for fall risk and evaluate those found to be at risk for falls, the CDC used the AGS/BGS guidelines to create the Stopping Elderly Accidents, Deaths, and Injury (STEADI) Toolkit (6). The STEADI toolkit was designed to help primary care providers incorporate older adult fall risk assessment, treatment, and referral into clinical practice and to facilitate patient referrals to community-based fall prevention programs. The STEADI includes an algorithm that details each step of the screening, assessment, and referral process (6). A key strength of the STEADI toolkit is its consideration of multiple risk factors for falls; however, guidance for screening and assessing feet- and footwear-related risk factors is limited. Specifically, the CDC algorithm assessment includes the directive to “Assess feet/footwear” (7) and general recommendations are provided in a separate document, the *Coordinated Care Plan to Prevent Older Adult Falls* (8). These recommendations include assessing feet for decreased sensation, presence of foot deformities, and use of footwear without good arch support, heel support, and sturdy soles with good grip (8).

A 2020 systematic review conducted by members of our research team identified eight factors regarding feet or footwear-related impairments or functional limitations that may increase older adults' fall risk: (1) Neuropathy and Sensory Impairment, (2) Foot Pain, (3) Foot or Ankle Orthoses, (4) Shoe or Footwear, (5) Foot Deformities, (6) Strength, (7) Range of Motion, and (8) Skin or Changes in Plantar Soft Tissues (9). The Neville et al. (9) systematic review reflected findings from the systematic reviews conducted by Aboutorabi et al. (10) and Menant et al. (11) that identified the association between inappropriate footwear and falls, as well as the systematic review and meta-analysis by Menz et al (12) which concluded that foot pain, hallux valgus, and lesser toe deformity are risk factors for falling. Subsequent to publication of the Neville et al. (9) review, James et al. (13) summarized changes in the aging foot and podiatric problems which may be more common in older patients based on their review of the literature and described a method for physical examination with a specific focus on the needs of older patients. In addition to concluding that foot disorders are associated with falls and reduced mobility, those authors concluded that foot examination is a vital component when evaluating older adults' mobility and falls. Because assessment of older adult' fall risk is a shared responsibility among health care professionals, a systematic and evidence-based approach to screening for feet or footwear-related influences on fall risk that can easily be incorporated into interprofessional team members' practice is needed.

We know of no comprehensive, clinically feasible screening tools that interprofessional healthcare providers can use to screen for feet or footwear-related impairments or functional limitations that may increase older adults' fall risk. Therefore, this study aimed to use a two-phase study design to develop a tool that compliments the STEADI algorithm and supports interprofessional health care providers in their efforts to screen for feet/footwear-related influences on fall risk among community-dwelling older adults identified at risk for falling.

## Materials and Methods

**Figure 1:**
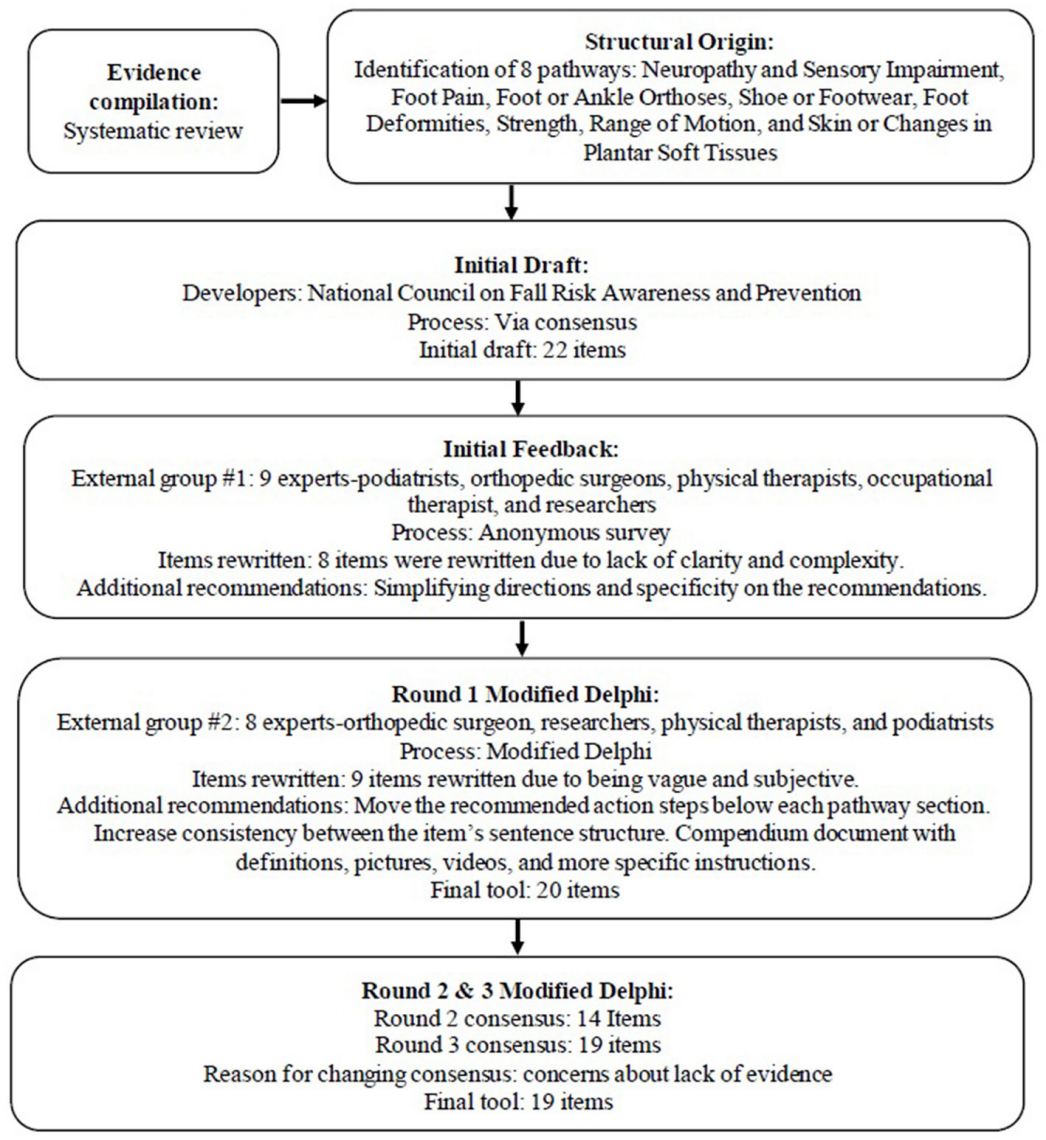
Flow diagram outlining Feet/Footwear-Related Fall Risk Screening Tool development methodology.

The study consisted of two phases. Both phases were informed by the Neville et al. (9) findings. The purpose of Phase 1 was to develop a strong initial draft of the screening tool. The purpose of Phase 2 was to refine the screening tool and examine the tool's content validity. Phase 2 of the study used a modified Delphi technique (14). The Checklist for Reporting Results of Internet E-Surveys (CHERRIES) was used to guide both phases of this study (15). Prior to Phase 1 data collection, the second author's Institution's Internal Review Board (IRB) deemed the study exempt from review and approved the study. Two different surveys were utilized in both Phase 1 and Phase 2. Informed consent was provided prior to participants starting the survey. Providing feedback and participating in the study was voluntary, and participants did not receive any incentives. Phase 1 and 2 data was collected anonymously using a secure online REDCap (Vanderbilt University; Nashville, TN) survey. For both phases, the survey items were developed by the authors, entered into REDCap by the second author (JV), and tested by the other authors of this study. REDCap was also used for all required communication. Participants were able to review and change their answers using the back button in all surveys throughout the study. For an outline of the tool's development process see Figure 1.

### Phase 1: Initial Screening Tool Development

#### Design and Procedure

The purpose of Phase 1 (January through October 2020) of the study was to generate a strong first draft of a screening tool that reflected the eight factors regarding feet or footwear-related impairments or functional limitations that may increase older adults' fall risk that were identified by Neville et al. (9). During Phase 1, the authors of the present study participated in a series of in-person and virtual meetings with other authors of the systematic review by Neville et al. (9) to accomplish the Phase 1 goal. Importantly, these Phase 1 meetings drew from the expertise of an interprofessional group of collaborators who were researchers and licensed professionals from the fields of podiatry, physical therapy, chiropractic medicine, and occupational therapy.

An iterative process was used to modify or eliminate questions that were double-barreled or redundant. The items were further refined to avoid double negatives, complex sentences, absolutes, or ambiguity. Because the goal was to create a screening tool that would accompany the STEADI, the members sought to format the tool like the STEADI, with a yes/no response format and “yes” answers triggering a recommended action to address the risk. To increase ease of use, the *Orthoses* and *Shoe/Footwear* categories were collapsed into one category, and the *Range of Motion* category was included with other items within the screening tool. Our development and review process led to an initial draft of the *Screening Tool for Feet/Footwear-Related Influences on Fall Risk*, a 22-item screening tool that consisted of six categories. The six categories were: *Footwear and Footwear Habits, Nail or Skin Changes, Foot and Ankle Deformities, Foot and Ankle Strength, Foot Pain*, and *Foot Sensation*. The screening tool also included recommended action steps for healthcare providers. The action steps were specific for each category and intended to support referral to healthcare providers with the expertise needed to address the problem identified. The authors of this study approved the initial screening tool and all subsequent changes.

The final part of Phase 1 involved gathering anonymous feedback on the screening tool from additional interprofessional experts in the field who were not previously involved in developing the tool. To generate a list of potential experts to participate in Phase 1, the co-authors of the Neville et al. (9) drew upon their extensive professional networks to identify master clinicians or researchers who had expertise in foot and ankle impairments and/or fall prevention that would inform their review of the screening tool. The final list of potential experts involved in Phase 1 was unanimously agreed upon by authors of the present study. All potential experts were invited to participate using an e-mail with a link to a REDCap survey. The participants' feedback was provided anonymously via a REDCap survey from April through May 2020. The survey contained 38 questions organized into sections. The experts were asked to rate each item on a 0% (do not agree) to 100% (strongly agree) scale. After each question, the experts had the opportunity to provide feedback in open-ended comments.

#### Phase 1 Analysis and Reporting

Prior to initiating data analysis, all data were checked for completeness. Descriptive analysis to examine percent agreement and ratings for questions related to the items and the scale's overall appeal and feasibility was completed using SPSS-Version 27 (IBM Corp, Armonk, NY). The authors revised the screening tool based on expert comments in anticipation of Phase 2.

### Phase 2: Modified Delphi Study

#### Design and Procedure

Phase 2 (October 2020 through February 2021) focused on refining the screening tool developed in Phase 1 using a modified Delphi study to evaluate the tool's content validity. A Delphi study is characterized by two or more rounds of questionnaires and controlled feedback; the specific methodologies depend on the type of technique selected (14). The present study employed a modified Delphi technique (14) that used online web surveys and pre-selected items based on Phase 1 findings. Phase 2 consisted of three rounds and aimed to: (a) further refine the screening tool and (b) evaluate the screening tool's content validity.

A new cohort of researchers and master clinicians were identified and recruited for Phase 2, using the same selection and recruitment procedures used in Phase 1. Nine of fourteen identified experts (herein referred to as “Delphi participants”) agreed to participate. This number of Delphi participants is consistent with recommendations by Trevelyan and Robinson (16). Delphi participants' data were excluded if they did not complete all three rounds of Phase 2.

##### Round 1

Delphi participants provided general feedback about the screening tool's items' clarity, quality, detail, and importance and were given the opportunity to recommend additional items. The survey for Round 1 contained 63 questions in total regarding (1) feedback on the overall format and instructions for the entire screening tool, (2) feedback on each item in the screening tool as in the pre-Delphi survey, and (3) feedback on the recommended actions presented in the screening tool with similar questions regarding clarity, detail, and importance. Round 1 results were used to refine the screening tool used for Rounds 2 and 3.

##### Round 2

For the final scale, participants were asked to rate the importance of including each item using a five-point scale (1 [strongly disagree] to 5 [strongly agree]), and based on responses, a per-item consensus was measured (see *Analysis* methods). The survey used for Round 2 contained 19 questions.

##### Round 3

Items that did not meet consensus during Round 2 were reassessed in Round 3. Each participant received a personalized document that provided the group's median and interquartile range for each item, along with the participants' original rating. With the individualized data document and their professional opinion, each expert was asked to either agree with the group or provide justification(s) about why they did not agree. Round 3 response options were the same as round 2.

#### Criteria and Rating of Questionnaire Items

The Delphi participants were instructed to apply different methodologies to rate the survey items in Round 1 than in Rounds 2 and 3. For Round 1, the Delphi participants appraised each item using three criteria: (1) clarity, (2) detail, and (3) the importance of the risk factor. For Rounds 2 through 3, they rated the items' importance of being in the final screening tool. All criteria used the same five-point scale (1 [strongly disagree] to 5 [strongly agree]). For all rounds, each question was followed by a free text box for the experts to comment about the items.

#### Phase 2 Analysis

All data were checked for completeness prior to analysis. Round 2 and 3 data were only used if the participant completed the entire survey. The *a priori* definition for including an item in the final screening tool was based on consensus, defined as ≥ 80% of the Delphi participants rating an item as strongly agree (5) or agree (4). While consensus for excluding was determined if > 80% of the Delphi participants rated an item as strongly disagree (1) or disagree (2) (17). Content validity was established using the Content Validity Index, which is based on a percentage of agreement > 80% (18). For the qualitative data provided in the comments, content analysis to detect and introduce new proposals and reformatting of items was completed and subjected to appraisal by the research team for consistency with the systematic review informing the screening tool (17).

## Results

### Phase 1 Results: Initial Screening Tool Development

All 22 items in the initial draft of the *Screening Tool for Feet/Footwear-Related Influences on Fall Risk* were kept, as they were deemed important by the internal and external group of participants. The external group of nine experts included three podiatrists, two orthopedic surgeons, two physical therapists, an occupational therapist, and a researcher. They recommended that the screening tool's instructions be simplified by explicitly identifying how the screening tool can be used with the STEADI. The experts rated the average overall clarity of the items as 81.2 (SD = 11.7) and provided feedback on eight items. The item with the lowest clarity (mean = 64.8; SD = 34.1) was within the foot strength section and dealt with forefoot raises to assess pretibial muscle (e.g., tibialis anterior) strength. The comments highlighted the lack of clarity about the alternation of the movements and the lack of standardized tools available in the literature to assess this. The item with the second-lowest clarity rating was related to skin changes (mean = 78.1; SD = 22.0). The recommendations were to define a “poor-fitting” shoe and when skin changes should trigger a recommended action to ameliorate fall risk. The other items that needed clarifying were the foot-pain and foot-deformity items.

Phase 1 experts also raised concern that the screening tool would result in too many positive risk factors and referrals. Part of this was due to a lack of clarity in the items; the reviewers thought that *all* patients with skin changes or flat feet are to be referred. However, only individuals first identified as being at risk for falling by the STEADI and then identified to have a feet- and footwear-related impairment are flagged for referral to appropriate healthcare providers (e.g., podiatrist or physical therapist). Therefore, the instructions on the screening tool were revised for clarification. The experts also recommended being specific about which providers should receive referrals in the recommended actions for items (e.g., include podiatrists and removed physical therapists in the “recommended actions” for older adults who have positive screenings in the *Nail and Skin Changes*).

The last set of Phase 1 recommendations from the experts focused on the screening tool's layout and included recommendations on improved workflow, length, and referral suggestions. The only recommendation regarding potential items to add was related to assessing gait and the use of an assistive device. Items pertaining to assessment of gait and use of assistive devices were not added to the screening tool for two reasons. First, the tool's focus is on screening for foot and footwear-related influences on fall risk. Second, the screening tool is intended to be complimentary to the STEADI algorithm which includes directives and guidelines for gait assessment. When asked about the screening tool's appeal and feasibility, the experts rated the average appeal as 79.1 (SD = 21.0) and the feasibility as 76.1 (SD = 21.0).

### Phase 2 Results: Modified Delphi Study

One of the initial nine Delphi participants did not complete all the study rounds and their data were excluded from the analysis. Because eight is still within the recommended number of participants for a modified Delphi, no additional steps were taken (16). The remaining eight experts consisted of an orthopedic surgeon, two researchers, two physical therapists, and three podiatrists, all with 10 to 35 years (mean = 19.3) of clinical and/or research experience.

#### Round 1 Findings

##### Instructions

The Delphi participants reported that the information in the instructions describing the relationship of the *Screening Tool for Feet/Footwear-Related Influences on Fall Risk* to the STEADI distracted from the tool's primary instructions and purpose. The feedback led to the following simplified directions, “*Impairments within the feet or poor footwear may be contributing to an ambulatory older adult's risk for falling. This screening tool was designed to screen for feet/footwear related influences for older adults who have been identified at risk for falling.”*

##### Layout

The Delphi participants suggested moving the recommended actions from a third column to a row at the bottom of each category. Participants' reasoning was to provide additional space to take notes, improve the delineation of sections, and improve ease of use. They also recommended increasing consistency between items' sentence structure (e.g., some items started with “patient,” and some did not) and to increase the consistency of the item stems within each category (e.g., the footwear category started with either “patient presents” or “patient reports” and was changed to “patient wears”).

##### Items

The Delphi participants recommended that objective values for items such as “poorly fitted shoes” or “high arch” and definitions for words such as “callus,” “corn,” and “bunion” be added. For the strength-related screening questions, they suggested more details about the directions and potential compensations. In order to incorporate the request for additional details and maintain the simplicity of the document, a compendium document was developed that includes information about the screening tool's development process, how to use the screening tool, safety considerations, definitions, directions for scoring each item, picture examples, links to videos, and references. For example, in the section on screening for foot deformities we have included definitions for hallux valgus, flat foot, high arches, contracted digits, and examples of other foot or ankle deformities. These definitions are followed by descriptions and photos of signs healthcare providers should look for when screening for foot deformities. More specifically, for contracted digits, we provided the following definition, “*deformities of the toes,”* followed by the description, “*toes that are not straight or flat on the ground,”* and pictures of examples, including hammertoes, claw toes, or mallet toes. It is important to note that the tool is meant for an interprofessional team and thus requires simple descriptions and directions that will be understood by all healthcare providers.

Delphi participants also suggested combining several related items (e.g., adding “callus” to “dry” or “hardened skin” and combining “skin irritation” and “ulcer”) with the rationale that the treatment recommendations are similar for the subgroup of items and reducing the number of items would make the screening tool more acceptable and feasible. Additionally, the Delphi participants recommended that an item related to ankle braces or orthoses be added. This recommendation is in line with the systematic review informing the tool (9); therefore, the authors of this study agreed with this recommendation. The following item was added to the scale, “*Wears or been advised to wear foot or ankle brace(s) or orthoses.”* These modifications led to the final 20-item screening tool.

### Rounds 2 and 3 Findings

**Figure 2:**
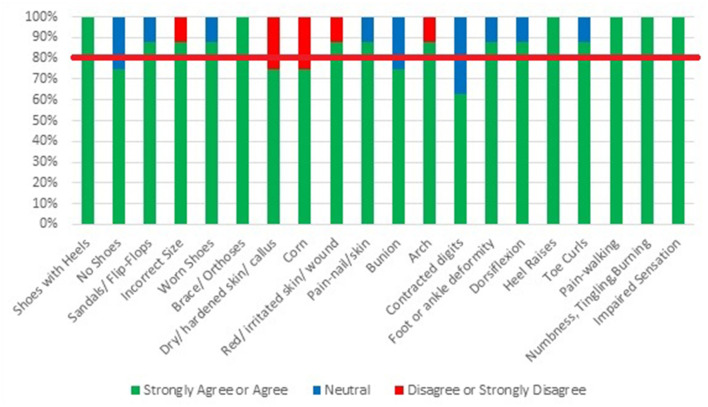
Results of modified Delphi-Round 2: expert opinion regarding the inclusion of items on the Feet/Footwear-Related Fall Risk Screening Tool.

**Figure 3:**
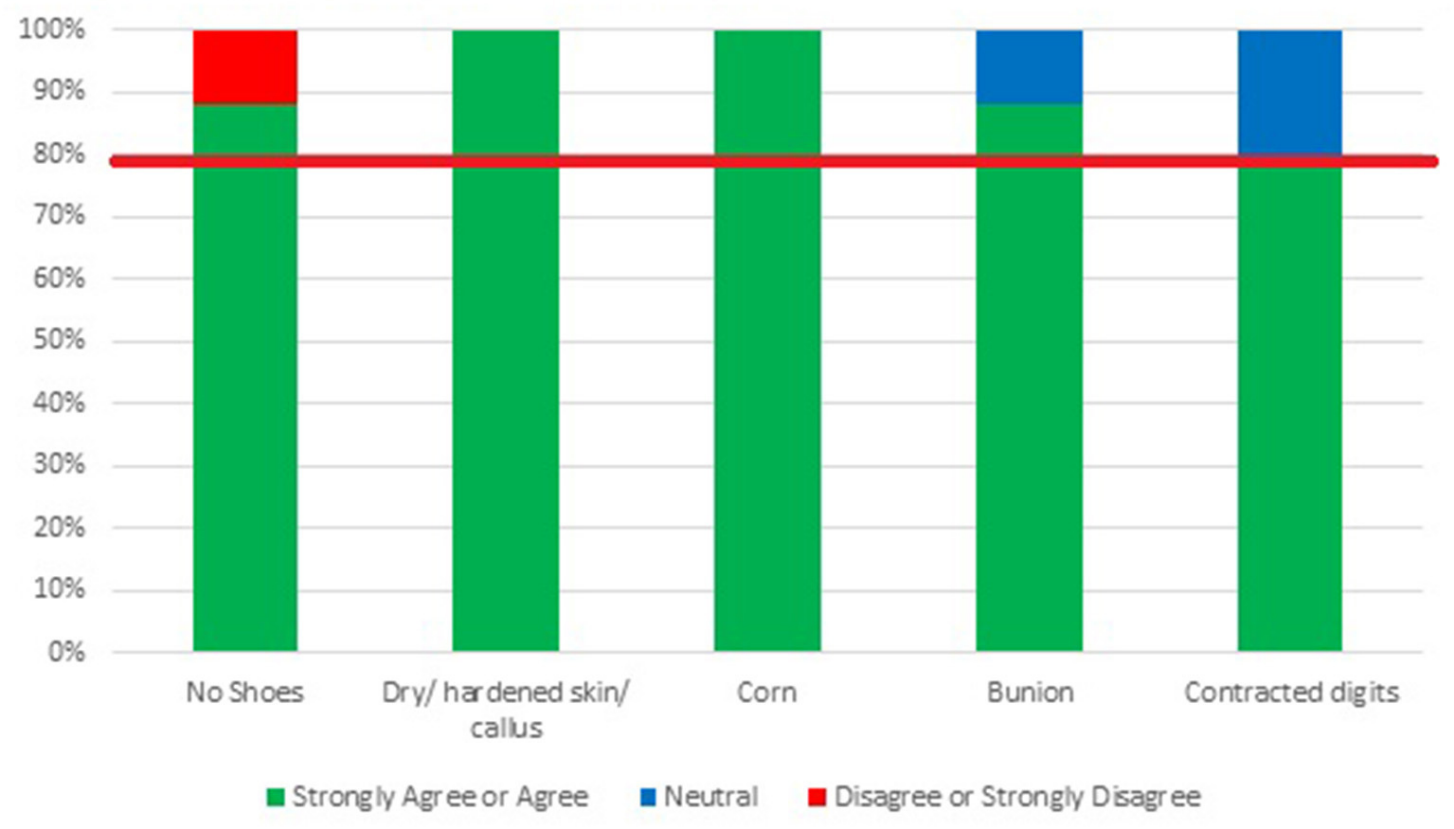
Results of modified Delphi-Round 3: expert opinion regarding the inclusion of items on the Final Feet/Footwear-Related Fall Risk Screening Tool. Items in this round were only assessed if the agreement was not met in Round 2.

Detailed results for Rounds 2 and 3 are presented in Figures 2, 3. In summary, during Round 2, Delphi participants met consensus on 75% of the items, with 15 items identified for inclusion in the final screening tool. After Round 3, Delphi participants were considered to have met consensus on all 20 items, indicating that the screening tool represents all facets of a given construct and thus, has content validity. One participant disagreed with the inclusion of the item “*walks barefoot or wears socks without shoes inside or outside the home”* in the final screening tool because they believed that “*barefoot (without socks) walking is much safer than wearing socks indoors”* but “*would endorse the question if it included wearing socks.”* As the item includes wearing socks as a risk factor, the screening tool developers felt that the participant might not have read the item thoroughly; therefore, the item was maintained in the final screening tool. According to the Delphi participants, the primary reason for changing an item from neutral to agree or strongly agree was related to evidence. Our conclusion is supported by the comment provided by Delphi Participant Eight, “*I had a 3 but was not familiar with specific evidence-based lit to support the idea.”* See Table 1 for a list of final items, examples of evidence supporting items and fall risk, and screening methods. For the final screening tool see Supplementary Material 1.

**Table 1:** The screening tool's items, examples of evidence supporting items and fall risk, and screening methods.

**Item**	**Examples of evidence supporting item selection**	**Screening methods**
Wears shoes with heels higher than 2.5 cm (1 in).	Compared to standing in standard shoes (2.5 cm or less), when standing in higher heels older adults have greater postural sway (19). Elevated heels have also been found to elicit increased double support time, heel horizontal velocity at heel strike, and toe clearance (19). Additionally, there are two systematic reviews that presented multiple studies that have identified the influence of elevated heels on stability and gait (9, 10).	Through observations and subjective questioning healthcare providers can identify what type of shoes their patients wear and/or if their patient has been asked to wear foot or ankle brace(s) or orthoses.
Walks barefoot or wears socks without shoes inside or outside the home.	Going barefoot increases the risk of falling [OR = 11.2; (20)] and among those who have a fall while wearing no shoes or wearing slippers there is an increased odds of sustaining a serious injury [OR = 2.27; (21)].	
Wears sandals, flip flops, and slippers.	Sandals may negatively affect postural stability (22) and wearing slippers at the time of a fall increases the odds of having an injury [OR = 2.27; (21)].	
Wears shoes that fit too tightly or too loosely.	In an outpatient geriatric clinic, 72% participants wore ill-fitting shoes. A larger percentage (56%) of individual who wore ill-fitting footwear reported a 6-month retrospective fall history compare to those who were appropriately fitted shoes [39% (23)].	
Wears or been advised to wear foot or ankle brace(s) or orthoses.	According to various cross-sectional and observational studies, it is hypothesized that foot or ankle brace (s) or orthoses enhance ankle stability and ankle proprioceptive feedback (24, 25), distribute pressure and maintain alignment (26), and enhance cutaneous mechanoreceptors (10). However, it is important to note that individuals who are advised to wear orthotics or braces may have an underlying diagnosis that increases their risk for falling, particularly if the brace is not fitting (9).	
Dry or hardened skin or callus.	Localized skin areas of hardness, callus, or corn can increase the risk of a person developing foot pain, an impairment associated with increased risk for falling (12, 27).	By visually inspecting patients' feet one can identify if the patient has any dryness, hardened skin, callus, corn, irritated skin, or a wound.
Corn.		
Red or irritated skin or wound.	Changes in the properties of soft tissues led to impaired gait and adaptation to irregular or uneven terrain, increasing an individual's risk for falling (28).	
Pain from any nail or skin changes in the feet.	Changes in nails or skin, including in-grown toenails, ulcers, or stasis dermatitis, can cause pain (29) and foot pain is associated with gait and balance impairments (12, 22).	Asking patients regarding their pain helps identify a potential increased risk for falling and identifying that the pain is secondary to nail or skin changes resulting in specific recommendations.
Bunion/Hallux valgus.	Individuals with history of falls have been identified to have more severe hallux valgus compared to non-fallers (12, 30, 31). Additionally, research has identified that individuals with hallux valgus had less lateral stability and greater coordinated stability errors (32)	A visual inspection of the foot and ankle can help screen for foot and ankle deformities.
Contracted digits.	Contractions make it difficult to recover from a loss of balance (33).	
Foot or ankle deformity.	Foot deformities are associated with kinetic and kinematic gait abnormalities (30, 31).	
Unable to complete 5 alternating forefoot raises while standing in 10 seconds.	Tibialis anterior is required for postural stability (33, 34).	A simple screen to see if a patient can perform the task repeatedly over a short period of time provides sufficient information to determine the need for further assessment.
Unable to complete 5 bilateral heel raises while standing in 10 seconds.	Gastrocnemius is required for postural stability (33, 34).	
Unable to curl toes.	Both Hallux and lesser toe weakness are associated with impaired balance performance (31, 34).	A simple screen to see if the patient can do it provides sufficient information to determine need for further assessment.
Currently reports foot pain that limits their ability to walk.	Compared to those without foot pain, individuals with foot pain have increased odds of falling [OR = 1.87–3.60; (12, 35)].	The impact of foot pain on walking can be determined by asking patients to rate their foot pain.
Currently reports numbness, tingling, or burning in the feet.	Among older adults who report falls, there is an association between higher plantar surface numbness and poorer balance as well as gait changes (36, 37). Additionally, plantar numbness is associated with a higher fear of falling, a risk factor of falling (37).	Asking patient about numbness, tingling, or burning provides insight about patients' sensation and potential need to do additional assessment.
Impaired light touch sensation on bottom of foot/toes.		Using either monofilament testing or performing the Ipswich test (38) provides insight about the patients' sensation and potential further actions.

## Discussion

To our knowledge, our *Screening Tool for Feet/Footwear-Related Influences on Fall Risk* is the first documented instrument to screen for feet or footwear-related impairments or functional limitations that influence fall risk among community-dwelling older adults. Its constructs and items reflect the eight categories of feet and footwear factors identified by Neville et al. that may increase fall risk in older adults (9). The tool is intended to be used after an individual is identified at risk for falls. The STEADI toolkit can be used to identify an individual at risk for falling by either completing the 12-question tool called “*Stay Independent”* or three key questions associated with increased prospective risk of falling (6). According to the STEADI algorithm, health care providers working with older adults who screen positive for fall risk are to “Assess feet/footwear” (7). The new tool developed through the present study supports interprofessional health care providers in their efforts to screen for feet/footwear-related influences on fall risk among community-dwelling older adults identified at risk for falling and refer older adults to the appropriate health care provider.

The importance of identifying modifiable fall risk factors is consistently highlighted in the fall prevention literature (5, 39). Further, feet and footwear-related influences on fall risk are consistently identified as an important part of a multifactorial assessment of fall risk factors. The *CDC's Coordinated Care Plan to Prevent Older Adult Falls* (8) provides general recommendations, including assessing feet for decreased sensation, presence of foot deformities, and use of footwear “with good arch support, heel support, and sturdy soles with good grip” but does not support systematic screening for foot/footwear influence on fall risk. The screening tool developed in this study is the first validated tool, to our knowledge, to guide healthcare providers in screening for feet- and footwear-related factors that may increase the risk of falling among community-dwelling older adults. The standardized screening methodology delineated via the tool will result in more effective and efficient screening processes (40), thus we hypothesize that our screening tool will facilitate implementation of feet and footwear screenings conducted in the context of clinically-based fall prevention efforts (41, 42).

The modified Delphi methodology utilized to create the new tool helps to ensure that items included in the tool are relevant and important to the scale administrators, thereby increasing the likelihood that the tool will be used in clinical practice. The study participants' high agreement on the items included in the final score helped confirm each item's relevance and importance and provides support for content validity. The recommendations describing referral options for older adults presenting with specific feet/footwear influences that increase the risk of falls are intended to foster action on healthcare providers' part (i.e., referral to other providers with the expertise needed) and subsequent targeted intervention.

The items selected for the final *Screening Tool for Feet/Footwear-Related Influences on Fall Risk* are further supported by James et al. (13) as they provided similar recommendations on categories of foot disorders that should be examined among older adults. These categories of foot disorder included the following: (1) nail disorder (i.e., ingrown toenails and fungal nail infections); (2) Skin Disorders (i.e., pigmented lesions, ulcers, xerosis, and hyperkeratosis); (3) Bone/Joint Disorders (i.e., foot pain or arthritis, hallux valgus, hallux rigidus, hammertoes, pes cavus/planus, and plantar fasciitis/heel pain); and (4) Neurovascular disorders (i.e., peripheral arterial disease and impaired light touch). However, it is important to note that falls was not a primary outcome, instead James et al. (13) focused on important foot disorders that need to be examined for a variety of reasons, including falls, pain, and lower limb ulcers.

This study has limitations that may affect the generalizability of findings. The experts represented on the Delphi panel included United States-based healthcare providers who may not represent care provided outside the United States based health-care system. Further, those at risk for falls are likely to come into contact with many providers including nurses and primary care physicians who could also be included in future studies. Self-selection bias and information bias may have occurred as a different cohort of experts with different perspectives may have rated these items differently. Finally, the items and domains within the scale are based on literature and multiple rounds of informal and formal feedback from experts, which are the recommended first steps in developing a scale (43). Future work involving factor analysis can be used to further inform the tool's construct validity.

Future studies investigating the tool's construct validity, predictive validity, and reliability via a large-scale validation study are needed. Furthermore, future studies will need to examine the implementation potential of the tool. Feedback from interprofessional health care providers on topics including time needed for administration and other influences on acceptability, such as opinions about the tool's content, may inform revisions that will enhance clinical utility. In addition to providing insights regarding the tool's acceptability among patients, feedback from older adults is needed to yield insights regarding older adults' priorities for foot and foot-wear related influences on fall risk. Such priorities may or may not be evidence-based but can be addressed as part of fall prevention education efforts.

A two-phase process, informed by a systematic review conducted by our team and including a modified Delphi study, was used to develop the *Screening Tool for Feet/Footwear-Related Influences on Fall Risk*. The tool, intended for use by interprofessional healthcare providers, is the first tool to screen for feet/footwear-related influences on fall risk among community-dwelling adults identified at risk of falling. A large-scale validation study is needed to gain further insight into the tool's reliability and validity, including test-retest reliability, internal consistency, construct validity, and predictive validity. Additionally, implementation research is needed to investigate how the new tool performs in clinical practice.

## Data Availability Statement

The raw data supporting the conclusions of this article will be made available by the authors, without undue reservation.

## Ethics Statement

The studies involving human participants were reviewed and approved by University of Arkansas for Medical Sciences. The participants provided their written informed consent to participate in this study.

## Author Contributions

MW ensured proper modified Delphi methodology was implemented, analysed the data, wrote the initial draft, and prepared the manuscript for submission. JV managed ethical considerations, the internal review board processes, recruitment, and data acquisition. All authors contributed substantially to the study design, recruitment, data acquisition and interpretation, draft revisions, and final approval of the manuscript.

## Funding

This project was supported by the Translational Research Institute (TRI), grants KL2 TR003108 (JLV) and UL1 TR003107 and UL1 TR003108 (UAMS) through the National Center for Advancing Translational Sciences (NCATS) of the National Institutes of Health (NIH). The content is solely the responsibility of the authors and does not necessarily represent the official views of the NIH.

## Conflict of Interest

The authors declare that the research was conducted in the absence of any commercial or financial relationships that could be construed as a potential conflict of interest.

## Publisher's Note

All claims expressed in this article are solely those of the authors and do not necessarily represent those of their affiliated organizations, or those of the publisher, the editors and the reviewers. Any product that may be evaluated in this article, or claim that may be made by its manufacturer, is not guaranteed or endorsed by the publisher.
