# Supplementary material for: Feet/Footwear-Related Fall Risk Screening Tool for Older Adults: Development and Content Validation
Source: Front Public Health. 2022 Feb 2;9:807019. doi: 10.3389/fpubh.2021.807019 (PMC8847373; doi:10.3389/fpubh.2021.807019)
Supplement: Supplementary file 1 [file Data_Sheet_1.PDF]

National Council on Fall Risk Awareness and Prevention

Screening Tool for Feet / Footwear Related Influences on Fall Risk

Impairments within the feet or poor footwear may be contributing to an ambulatory older adult's risk for falling. This screening tool was designed to screen for feet/footwear related influences for older adults who have been identified at risk for falling.

Screening Focus	Present?		Notes
**Screening for Footwear and Footwear Habits**			
Wears shoes with heels higher than 2.5 cm (1 in)	☐ Yes	☐ No	
Walks barefoot or wears socks without shoes inside or outside the home	☐ Yes	☐ No	
Wears sandals, flip flops, slippers	☐ Yes	☐ No	
Wears shoes that fit too tightly or too loosely	☐ Yes	☐ No	
Wears shoes with worn soles, heels or treads	☐ Yes	☐ No	
Wears or been advised to wear foot or ankle brace(s) or orthoses	☐ Yes	☐ No	
**Recommended Action -** Patients should be encouraged to always wear shoes with low heels and firm, slip-resistant soles, both inside and outside the home. Advise patients that going barefoot or only wearing socks increases their chances of falling. High collared shoes, i.e. shoes with openings at the ankle, may improve position sense and stability. If footwear problems are evident, refer the patient to a podiatrist or certified pedorthist.			

**Screening for Nail or Skin Changes**			
Dry or hardened skin or callus	☐ Yes	☐ No	
Corn	☐ Yes	☐ No	
Red or irritated skin or wound	☐ Yes	☐ No	
Pain from any nail or skin changes in the feet	☐ Yes	☐ No	
**Recommended Action**<br>For any "Yes" item in this section consider referral to foot and ankle specialist (e.g., podiatrist) as appropriate.			

Copyright © 2020 National Council on Fall Risk Awareness and Prevention. All rights reserved

Screening Focus	Present?		Notes
**Screening for Foot and Ankle Deformities**			
Bunion / Hallux valgus	▢ Yes	▢ No	
Flat foot or high arch	▢ Yes	▢ No	
Contracted digits	▢ Yes	▢ No	
Foot or ankle deformity	▢ Yes	▢ No	
**Recommended Action**<br>For any “Yes” item in this section consider referral to foot and ankle specialist (e.g., orthopedic surgeon, physical therapist, podiatrist) as appropriate.			
**Screening for Foot and Ankle Strength**			
Unable to complete 5 alternating forefoot raises while standing in 10 seconds	▢ Yes	▢ No	
Unable to complete 5 bilateral heel raises while standing in 10 seconds	▢ Yes	▢ No	
Unable to curl toes	▢ Yes	▢ No	
**Recommended Action**<br>For any “Yes” item in this section consider referral to foot and ankle specialist (e.g., physical therapist) as appropriate.			
**Screening for Foot Pain**			
Currently reports foot pain that limits their ability to walk	▢ Yes	▢ No	
**Recommended Action**<br>For any “Yes” item in this section consider referral to healthcare provider (e.g., podiatrist, geriatrician or primary care physician) as appropriate.			
**Screening for Foot Sensation**			
Currently reports numbness, tingling, or burning in the feet	▢ Yes	▢ No	
Impaired light touch sensation on bottom of foot / toes	▢ Yes	▢ No	
**Recommended Action**<br>For any “Yes” item in this section consider referral to a foot and ankle specialist (e.g., podiatrist) as appropriate.			

Copyright © 2020 National Council on Fall Risk Awareness and Prevention. All rights reserved
